# Continuous capture of recombinant antibodies by ZnCl**_2_** precipitation without polyethylene glycol

**DOI:** 10.1002/elsc.201900160

**Published:** 2020-03-24

**Authors:** Gregory Dutra, Daniel Komuczki, Alois Jungbauer, Peter Satzer

**Affiliations:** ^1^ Department of Biotechnology University of Natural Resources and Life Sciences Vienna Austria; ^2^ Austrian Centre of Industrial Biotechnology (ACIB) Vienna Austria

**Keywords:** precipitation, downstream processing, IgG, zinc, purification

## Abstract

The capture of recombinant antibodies from cell culture broth is the first critical step of downstream processing. We were able to develop a precipitation‐based method for the capture and purification of monoclonal antibodies based on divalent cations, namely ZnCl_2_. Traditional precipitation processes have to deal with high dilution factors especially for resolubilization and higher viscosity due to the use of PEG as precipitation or co‐precipitation agent. By the use of the crosslinking nature of divalent cations without the use of PEG, we kept viscosity from the supernatant and resolubilization dilution factors very low. This is especially beneficial for the solid–liquid separation for the harvest and wash of the precipitate in continuous mode. For this harvest and wash, we used tangential flow filtration that benefits a lot from low viscosity solutions, which minimizes the membrane fouling. With this precipitation based on ZnCl_2,_ we were able to implement a very lean and efficient process. We demonstrated precipitation studies with three different antibodies, Adalimumab, Trastuzumab, and Denosumab, and a continuous capture case study using tangential flow filtration for precipitate recovery. In this study, we achieved yields of 70%.

**Abbreviations**
CDRs: complementarity‐determining regions
FBRM: focused beam reflectance measurement
IgG: immunoglobulin G
PEG: polyethylene glycol
pI: isoelectric point

## 1 INTRODUCTION

Precipitation is a notably flexible method that has been extensively used to reduce process volumes, fractionate, and separate products from complex mixtures. More recently, precipitation has been implemented in the capture step of monoclonal antibodies [1, 2, 3, 4, 5, 6, 7]. Besides its robustness, high yield, purity, and recovery rates, it allows a good transition from a batch to a continuous operation mode with a relatively simple set up [1, 3]. Additionally, a continuous recovery of the precipitate using a set of sequential tangential flow filtration units was enabled [4]. The results obtained in terms of yields and purities are comparable with the standard Protein A chromatographic capture step [4]. To achieve this, polyethylene glycol (PEG) was used as the main precipitant agent [4, 6, 8, 9, 10, 11]. It is a simple approach, operated at room temperature, with fast kinetics [12]. Nevertheless, the addition of PEG to liquid mixtures increases its viscosity. Regarding downstream processing, this increase in viscosity can result in some drawbacks, especially in the case of membrane filtration where it leads to a lower flux through the membrane and increased system pressure. Burgstaller and co‐workers [4] already showed that adding ZnCl_2_ to the mixture reduces considerably the amount of PEG required to selectively precipitate most of the antibodies [13].

Metal ions selectively precipitate proteins from solution by binding to the amino acid side chains. The metal ion bridges to other protein molecules, cross‐linking the monomers creating protein clusters. This reduces the protein solubility leading to precipitation [12, 13]. Previous authors reported that Zn^2+^ and other metal cations have the propensity to form metal‐protein complexes [11, 12, 13, 14, 15]. Copper with insulin [13], calcium with casein [13], Zinc with recombinant human growth factor and nerve growth factor [16] are some examples. In the case of immunoglobulins, Zn^2+^ has shown to form relatively stable complexes by binding to histidine and cysteine residues on the protein surface, possibly through exposed imidazole and thiol groups, respectively [15]. Przybycien and Iyer demonstrated through experimental data and model simulations that an increased number of metal ion binding sites on the protein surface increases the rate of aggregation [13, 14]. According to their study, immunoglobulins were found to have much lower aggregation time constants than the corresponding concentrations of albumin [14]. Besides the rate of aggregation, Gill and colleagues [13] reported that Zinc concentrations above 3 mM produced heavier precipitates with enhanced settling properties and CHO cell proteins are precipitated to a greater degree at low Zinc concentrations when compared to yeast proteins.

In this present study, we focus on the antibody precipitation with the use of a divalent metal cation, Zn^2+^ without addition of PEG, aiming to get a better picture of how the use of this metal can help improve and simplify the capture step of immunoglobulins.

## 2 MATERIALS AND METHODS

### 2.1 Cell culture

IgG1, Adalimumab (pI 8.2) was produced in Chinese hamster ovary (CHO) cells in fed‐batch fermentation. Trastuzumab (pI 8.4) was also produced in CHO cells but a perfusion system. IgG2 Denosumab (pI 8.3) was bought from Amgen (California, USA). For primary clarification, cells were removed by centrifugation and the host cell broth was filtered with a 0.22 µm membrane (Merck KGaA, Darmstadt, Germany), having, in the end, a host cell clarified broth (HCCB).

### 2.2 Precipitation optimization

Before testing, the cell culture broth pH was measured. All experiments were performed in 96‐deep‐well plates. Dilutions from 1 to 12 mM of ZnCl_2_ were prepared from a 100 mM solution and then added to 0.5 mL of the cell culture broth containing the antibody. After 20 min of incubation at room temperature on the end‐over‐end shaker (Stuart rotator SB3; Cole‐ Parmer, Vernon Hills, IL), the plate was centrifuged at 4000 rcf for 10 min (Centrifuge Heraeus Multifuge X3, Rotor HIGHPlateTM 6000; Thermo Fisher Scientific, Waltham, MA). The supernatant was withdrawn and filtered through 0.2 µm filters (0.2 µm GHP AcroPrepTM 96 filter plate; Pall Life Sciences, Ann Arbor, MI) and analyzed with protein A affinity chromatography (described in Section 2.3).

**PRACTICAL APPLICATION**

With the present study, we were able to show that the capture of recombinant antibodies with only a ZnCl_2_ precipitation‐based method is an improvement of previous ones.

By removing the use of PEG, the precipitate solution viscosity was maintained allowing the use of higher flow rates in filtration units without substantial increase in the transmembrane pressure; reducing the process overall cost and environmental footprint. Additionally, precipitates are stable during the wash step with no product loss and precipitate solubilization requires less volume maintaining the impurities clearance.

The process robustness makes it scalable and a continuous mode of operation can be easily set up.

### 2.3 Protein A chromatography

HPLC Protein A affinity chromatography was used to determine the antibody concentration. We used a Dionex UltiMate 3000 HPLC system equipped with a diode array detector (Thermo Fisher Scientific). Mobile phase A was 50 mM phosphate buffer, pH 7.0. Mobile phase B was a 100 mM glycine buffer, pH 2.5. Before usage, all buffers were filtered through 0.22 µm filters (Merck KGaA) and degassed. The system was run at a flow rate of 2.5 mL/min. We loaded 20 µL of the sample, filtered, on a POROS A 20 µm Column (2.1 × 30 mm, 0.1 mL; Thermo Scientific). The column was equilibrated with 10 column volumes of mobile phase A, eluted with a step gradient with 20 column volumes of 100% mobile phase B, and re‐equilibrated with 30 column volumes of mobile phase A. The absorbance at 280 nm was measured. We used a similar protein A purified IgG_1_ as the calibration standard. The calibration range was 0.1–8 mg/mL. We evaluated and quantified the results with the Chromeleon™ 7 software (Thermo Fisher Scientific).

### 2.4 Size exclusion chromatography

We used size exclusion chromatography to estimate product purity. For HPLC analytics, we used a Dionex UltiMate 3000 HPLC system equipped with a diode array detector (Thermo Fisher Scientific). The running buffer was a 50 mM potassium phosphate buffer with 150 mM NaCl, pH 7.0 (Merck KGaA). The buffer was filtered through 0.22 µm filters (Merck KGaA) and degassed. We applied 20 µL of the filtered sample to a TSKgel® G3000SWXL HPLC Column (5 µm, 7.8 × 300 mm) in combination with a TSKgel SWXL Guard Column (7 µm, 6.0 × 40 mm; Tosoh, Tokyo, Japan). The absorbance at 280 nm was recorded, and the results were evaluated with the Chromeleon™ 7 software (Thermo Fisher Scientific). The antibody purity was calculated as the ratio of the monomer peak area (retention time 21.2 min) to the sum of all peak areas, based on the 280 nm signal.

### 2.5 Particle size distribution measurements

To measure the particle size distribution, we used the Particle Track G400 probe equipped with Focused Beam Refractance Measurement (FBRM) technology (Mettler Toledo). We started by filling the EasyMax 102 reactor (Mettler Toledo) with 70 mL of filtered HCCB. The reactor mixing was set to 200 rpm, pH, and temperature were recorded. The probe was then mounted at an angle of 30° to the vertical axis and we started recording. The recording settings were 2 m/s, chord length selection of Primary (fines), on a chord length ranging from 0 to 100 µm. After 5 min of equilibration, the desired amount of a stock solution of ZnCl_2_ was added and the pH was corrected with sodium hydroxide or hydrochloric acid. The size distribution change was recorded for 25 min. The iC FBRM (Mettler Toledo) software was used to record and treat the data. After the recording, the data were normalized using the program function applying the no weight statistics option.

### 2.6 Wash and resolubilization

For washing and resolubilization, preliminary tests were performed with 96‐well plates. After the precipitation and centrifugation, the supernatant was removed, and the same volume of the desired washing buffer was added. The precipitate was homogenized in the solution with up and down pipetting. After, the samples were centrifuged once more at 4000 rcf for 5 min (Centrifuge Heraeus Multifuge X3, Rotor HIGHPlateTM6000 (Thermo Fisher Scientific, Waltham, MA). The supernatant was once more removed and the same volume of resolubilization buffer was added.

### 2.7 Viscosity determination

To determine samples viscosity, we used the DV‐II +Pro viscometer (Brookfield Engineering Laboratories, Middleboro, MA, USA) with the cone spindle CPA‐40Z. The equipment was calibrated according to the instructions provided by the supplier. We analyzed the samples by adding 0.5 mL in the middle of the sample cup. The measurements were performed at room temperature, varying the shear rate from 75 to 525 s^−1^. Nonetheless, only the measurements with the Torque between 10 and 100% were accepted.

### 2.8 Continuous precipitation

Continuous precipitation was conducted in a self‐assembled tubular reactor setup. Spirally arranged standard lab tube, with approximately 3 m (Tygon® R‐3603, 4.8‐mm inner diameter; Saint‐Gobain, Courbevoie, France) filled with static mixers (HT‐40‐6.30‐24‐AC; Material Acetal; Stamixco AG, Wollerau, Switzerland) was vertically stacked. The setup was connected with polycarbonate Luer fittings (Cole‐Parmer). The cell culture broth was continuously pumped at a flow rate of 8 mL/min with a peristaltic pump into the tubular reactor. This was later combined with a feed stream of 0.5 M ZnCl_2_ pumped at a flow rate of 0.25 mL/min with a syringe pump and a feed stream of 0.1 M NaOH pumped at a flow rate of 0.2 mL/min with a second syringe pump. This ratio resulted in precipitation conditions of 12 mM ZnCl_2_ pH 7. The retention time on the tubular reactor was approximately 3 min at this flow rate and at last the tubular reactor as connected to one Äkta flux S system (GE Healthcare). The precipitate was continuously fed into the filtration system and after 30 min of continuous precipitation, the filtration system started processing the precipitate. It was concentrated in a 0.2 µm hollow fiber module (GE Healthcare) with a filter area of 50 cm² on a feed flow rate of 120 mL/min and a permeate flow rate of 8 mL/min. The retentate is recirculated into a tank, but the tank is continuously fed from the upstream process, and continuously bled to harvest concentrated precipitate. The ratio of feed to bleed (harvest) is 1:10. Afterward, the same membrane and settings were used in stage 2 for washing the concentrated precipitate.

### 2.9 dsDNA concentration

The concentration of dsDNA was measured using the Quant‐iT Picogreen dsDNA assay kit (Life Technologies, Carlsbad, CA, USA) in a 96‐well format according to the manufacturer's instructions. In brief, serial dilutions of samples and λ DNA standard were performed in a 96‐well plate in 1 × TE buffer (10 mM Tris‐HCl, 1 mM EDTA, pH 7.5). 100 µL of all dilutions were transferred to a fluorescent black bottom 96‐well plate, 100 µL of prediluted color reagent was added to each well and the signal intensities were measured in a plate reader using an excitation wavelength of 480 and an emission filter of 520 nm with a bandwidth of ± 20 nm each.

### 2.10 Host cell protein

A MaxiSorp Immuno 96‐well plate (NUNC, Roskilde, Den‐ mark) was coated with goat anti‐CHO HCP antibody (3G‐0016‐AF, Cygnus, Southport, NC, USA). Residual binding sites were blocked by incubation with 3% BSA (Sigma Aldrich) in TBS. A standard curve was prepared in duplicate for each plate using CHO HCP standard (F553H, Cygnus). Samples and pre‐diluted CHO HCP standard were serial 1:2 diluted with 1% BSA in TBS and transferred to the measurement plate. After incubation with goat anti‐CHO HCP antibody conjugated to HRP (1:2000 diluted in 1% BSA in TBS + 0.05% Tween 20), the TMB Peroxidase EIA Substrate Kit (Bio‐Rad, Hercules, USA) was used for staining. The enzymatic reaction was stopped with 1 N sulfuric acid. The absorbance at 450 nm was measured using a Tecan F500 Infinite plate reader (Tecan, Maennedorf, Switzerland).

## 3 RESULTS AND DISCUSSION

### 3.1 Precipitation

**FIGURE 1:**
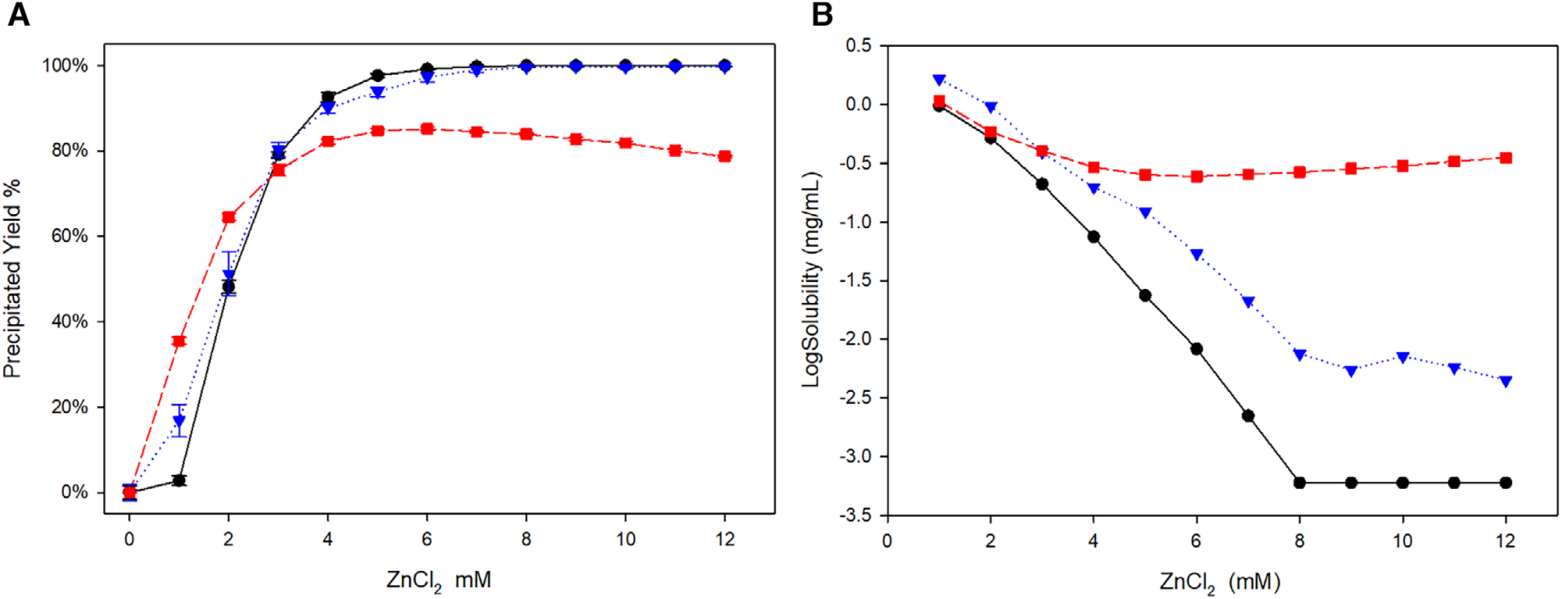
(A) Precipitations yields varying the ZnCl_2_ concentration for different antibodies. (B) Solubility curve for the increasing ZnCl_2_ concentrations. Dilutions were made from an aqueous 0.1 M ZnCl_2_ stock solution. Black circles Adalimumab, blue triangles Trastuzumab and red squares Denosumab

As stated, precipitating recombinant antibodies with zinc chloride is not a new principle. Compiling published data has shown that the combination of ZnCl_2_ and PEG resulted in a high yield (>90%) capture of recombinant antibodies [4]. More interestingly, some limited data can be found where a similar result was achieved when using ZnCl_2_ without PEG [7]. To avoid the issue of viscosity and high dilution factors for a precipitation‐based capture method for recombinant antibodies, we precipitated different antibodies using only ZnCl_2_. A crucial and often neglected part of precipitation studies is the solubilization of the precipitated antibody, which is crucial for a functional capture step and in this study, we developed a pH‐based resolubilization with low dilution factors. A high‐throughput protocol using microtiter plates was used to screen for the best conditions for precipitation as well as for solubilization. In an initial preliminary test, we determined the optimum amount of ZnCl_2_ to add to the clarified culture supernatant (Figure 1). At this stage we decided to keep the protocol as simple as possible, trying to avoid prior dilutions and pH alterations.

Zinc crosslinks protein monomers creating a bigger protein cluster that leads to precipitation. The initial test showed that 6 mM of ZnCl_2_ is enough to precipitate most of Adalimumab and Trastuzumab. Nonetheless, for Denosumab the highest yield obtained was around 80% with the same cation concentration. Additionally, further addition of Zn^2+^ resulted in even lower yields. These results show that the cation concentration does not seem to be the limiting factor in the case of Denosumab. When compared to other proteins, immunoglobulins have a higher precipitation rate, which is attributed to the higher number of surface‐exposed histidines favoring the protein cross‐linking [15]. In this case, comparing the three different recombinant antibodies of the class IgG the lower yield of Denosumab could be due to less surface exposed histidines, but the yield decrease with the further addition of ZnCl_2_ cannot be explained by this argument. We can compare the amino acid sequence of the three different antibodies, but one should regard that a different number of histidines in the protein sequence does not necessarily mean that those are available at the protein surface. Wang et al. reported that most of the commercially available IgGs have an amino acid sequence similarity above 85% [17]. Also, the majority of commercially available antibodies share the same protein backbone and the most diverging zones are the Complementarity‐determining regions (CDRs) and the hinge regions in the case of different IgG subclasses [17].

Simulations presented by Iyer and Przybycien showed that besides the metal‐binding sites on the protein surface, precipitation is also highly dependent on the pH of the solution and the pKa of the metal–ligand complex [14]. Protons compete effectively with the metal ions via a mass action effect reducing the number of sites available for metal ion binding. Zinc among other cations causes strong pH effects by hydrolysis [18]. In the present case, with the addition of ZnCl_2_, there is a pH shift from 7.2 down to 6 when 12 mM salt is added. This could be an indication of why we have lower yields with 12 mM compared to 6 mM for Denosumab, as it is not due to the addition of ZnCl_2_ itself but due to the change in pH caused by the addition of salt.

**FIGURE 2:**
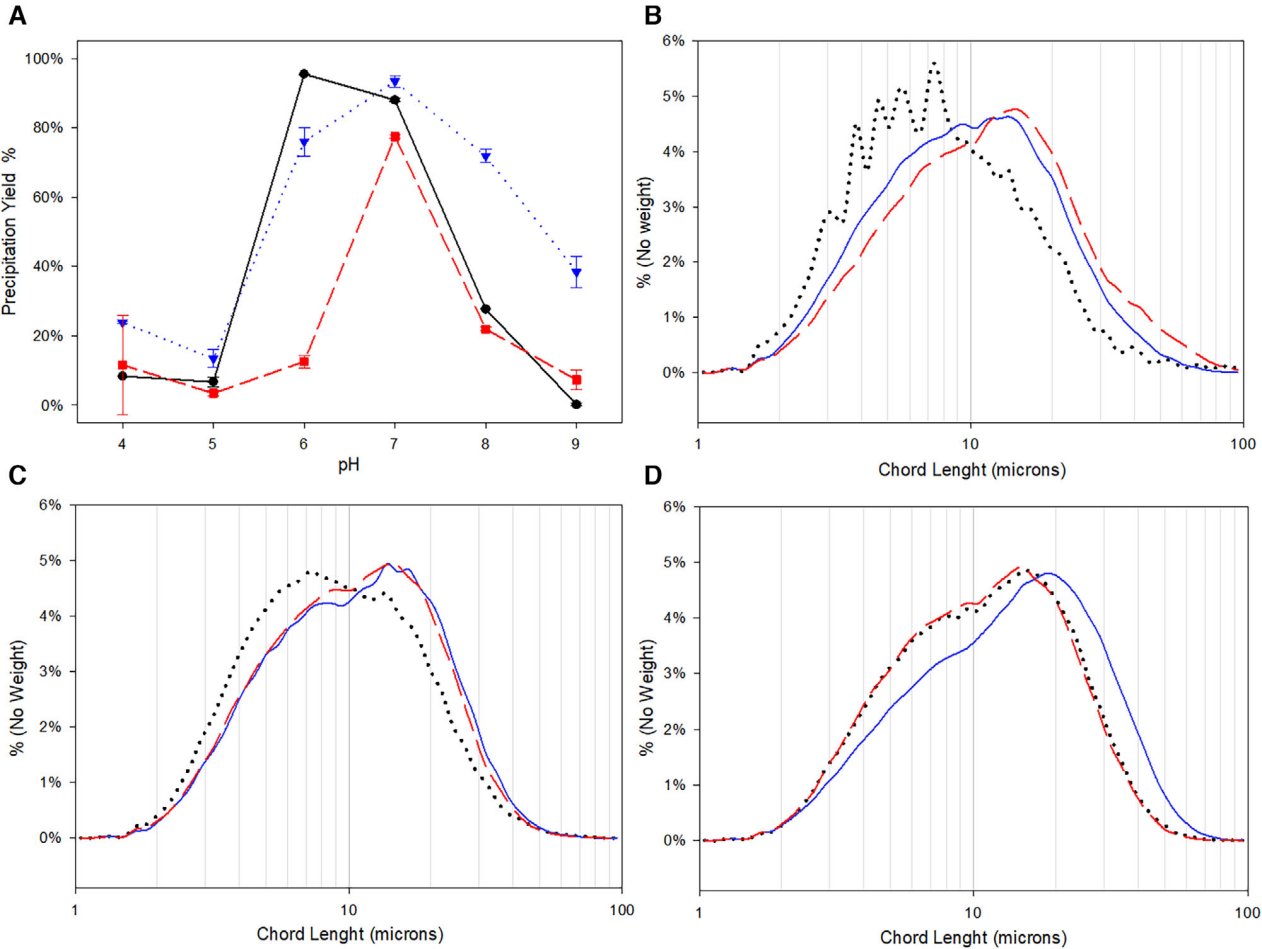
(A) Precipitation yields varying the pH for different antibodies. Black circles Adalimumab, blue triangles Trastuzumab and red squares Denosumab. B‐C‐D clusters size distribution for Adalimumab, Trastuzumab and Denosumab respectively. Black dotted line pH 8, blue solid line pH 7 and red dashed line pH 6. HCCB pH changes were performed using stock solutions of HCl or NaOH

To quantify the effect of pH, precipitation maintaining the 12 mM of ZnCl_2_ but varying the pH were carried out and yields are shown in Figure 2A. At the same time, the precipitation was monitored with Focused Beam Reflectance Measurement (FBRM) and the protein cluster size distribution at different pH is also presented in Figure 2B–D. As it seems, the optimum pH for the precipitation is around pH 6 and 7 for all the recombinant antibodies. The most specific case is the Denosumab, only having high precipitation yields at pH 7. According to the FBRM data, the different pH also has a great impact on the size of the precipitate clusters, as to be expected. For each case, the pH condition that generates the biggest clusters are also the ones having better yields. In an industrial setup, this also means that the FBRM in‐line monitoring can be used to estimate the resulting yields without offline measurement of the actual concentration during precipitation.

As discussed before, protons actively compete with the metal ion for the binding site. That explains the lower yields and the decreased cluster size at lower pHs (4 and 5), but not the lower yields at high pH (8 and 9). The difference between pH 6 and 7 for Adalimumab and Trastuzumab is not as pronounced as in the case of Denosumab, suggesting that they are less sensitive at this range. Histidine residues are known to dominate the titration behavior of proteins in the pH 6 (pKa of the side chain) to 7.6 (pI) range [13, 18]. That could be an indication that Denosumab has fewer binding sites compared to the other IgGs. Nonetheless, one should also regard that a different pH also means a different protein surface net charge. The interaction between two proteins negatively charged residues bridged by the cation is induced by a short‐range attraction. Thus, the aggregation of the proteins is also dependent on the balance between short‐range attractive forces and long‐range repulsive forces. That can create the misconception that the protein precipitation would be more effective at the protein pI, also in the case of Zn^2+^ as has been reported for many other precipitations like PEG precipitation without salt addition. Most of the commercial antibodies have the pI around pH 8–9, which is also the case for the antibodies in this study. As we can see in the Figure 2A, the optimum pH for precipitation is considerably below the pI, which means that we are not recreating a simple isoelectric precipitation when using ZnCl_2_, but that we have a different mechanism of precipitation connected more to the charge of histidines and less to the overall charge of the antibody. This is explained by the data presented by Schriber and co‐workers [17, 19, 20] as well as the data in our study. They have shown that the binding of the multivalent ion leads to a weakened protein surface charge and a subsequent protein condensation. As the metal ion binds to the protein, the surface charge becomes more positive. This is beneficial to the protein condensation to a certain limit. Saturating the mixture with the bridging ion results in the inversion of the protein charge surface due to an extensive binding of the cation. Consequently, this saturation also inhibits the cross‐linking. Summing up, the pH has a direct effect on the precipitation because of the competition between the proton and the cation for the binding site on the protein surface, and an indirect effect since it also modulates the protein surface charge.

**FIGURE 3:**
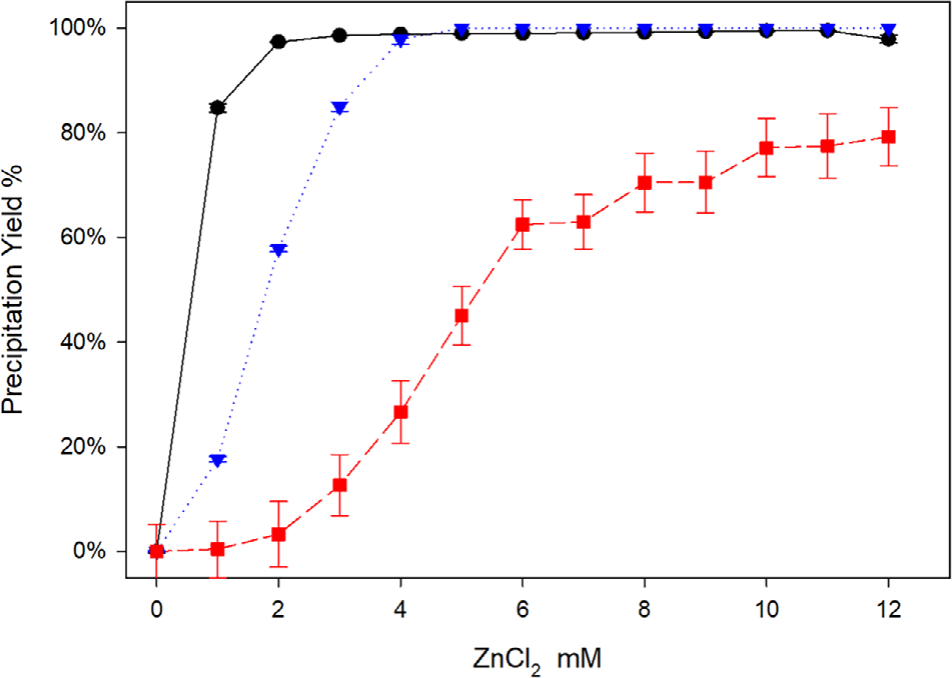
Precipitations yields varying the ZnCl_2_ concentration for different antibodies adjusting the pH. Black circles Adalimumab pH 6, blue triangles Trastuzumab pH 7 and red squares Denosumab pH 7

Taking all this data into consideration, a new set of precipitation curves were performed where we ensured a constant pH for Adalimumab at pH 6, Trastuzumab and Denosumab at pH 7. (Figure 3). With the optimized conditions, we can see an increase in the precipitation yield with the increase of ZnCl_2_ concentration, but still, the highest yield for Denosumab was around 80% as before. A further increase in the salt concentration resulted in the same yield.

Adalimumab and Trastuzumab are an IgG_1_ and Denosumab is an IgG_2_. Differences in the IgG subclasses are well known. The main difference between IgG 1 and 2 is in the hinge region [22]. The length and flexibility of the hinge region vary with the IgG subcases, where IgG_1_ is one of the most flexible and IgG_2_, on the other hand, having the shorter hinge and therefore being less flexible [21, 22]. Hinge regions of IgG_2_ are more rigid due to a poly‐proline helix, stabilized by up to four chain disulfide bridges, which is the case of Denosumab [24]. This lack of flexibility may help explain the lower yields in the precipitation. A rigid molecule is more likely to hinder binding sites and restrict the cross‐link due to electrostatic repulsions. Going even further and comparing the Adalimumab, Trastuzumab, and Denosumab CDRs, the last, is the only one in this group that does not have a histidine residue on the CDR. So, having possibly fewer binding sites than the other two IgGs and those binding sites being less accessible due to steric hindrances and less flexibility could explain the lower yield seen in Denosumab. While this is a possible explanation, a deeper investigation will be needed to accurately validate this statement. Nonetheless, one should bear in mind that little changes can cause a major effect.

### 3.2 Wash and resolubilization

For a successful precipitation capture step, the harvest, wash, and resolubilization of the precipitate is a major factor to be considered that is often neglected by precipitation studies. Taking the discussed mechanism of ZnCl_2_ precipitation into account, we designed a resolubilization based on a pH shift to low pH, effectively negating the binding site at the Histidine by protonating it below the pKa of the side chain. Also, to ensure good removal of impurities, a wash step prior to resolubilization was added. For the wash step, we used a 50 mM Tris‐HCl pH 7 buffer for washing without the addition of ZnCl_2_ in the washing buffer. Preliminary tests on a small scale showed that our product will remain in precipitated form unless the pH drops below 6 showing a very strong binding between protein and cation. No loss of antibody was observed due to the wash for any of the three antibodies. Regarding the resolubilization, we screened different buffer compositions (data not shown) and the best recoveries were obtained with a 1:1 dilution with a 100 mM sodium acetate pH 5 (or below) buffer.

As a capture step, the main goal is to isolate the product from most of the impurities and to concentrate the product as much as possible, or at least avoid the product dilution commonly found in precipitation based purification with dilution factors of up to 10 depending on the conditions [1, 4]. In this case, we were able to recover most of the Adalimumab and Trastuzumab after precipitation, wash, and resolubilization, the yields were above 90% while the yield for Denosumab was significantly lower, approximately 73%. Impurities were removed 7.6‐fold, which is comparable to the eightfold reduction presented by Burgstaller et al. and Li et al. but avoiding the larger dilution factors. With our setup, the final sample has the same initial volume and a low salt concentration that is preferable for subsequent polishing steps typically based on ion‐exchange chromatography. This setup is a significant improvement from the capture and resolubilization presented by Burgstaller and also Li [4, 7].

### 3.3 Sample viscosity

**FIGURE 4:**
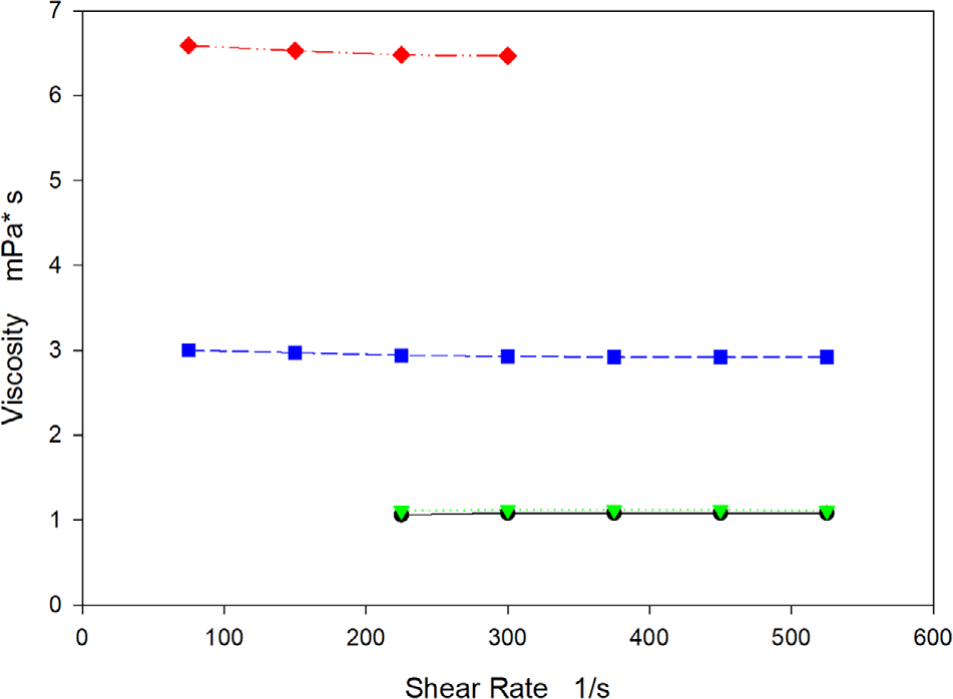
Viscosity of HCCB containing Trastuzumab before and after antibody precipitation. Black circles HCCB, green triangles antibody precipitated with 12 mM ZnCl_2_, blue squares antibody precipitated with 7% PEG6000 and 2 mM ZnCl_2_, red diamonds antibody precipitated with 14% PEG6000

One of the great advantages of not using PEG is the prevention of a considerable increase in the sample viscosity. We performed viscosity measurements on samples processed by three different methods. The method from Sommer et al. [2] using 14% of PEG6000, the method from Burgstaller et al.[4] combining 7% of PEG6000 and 2 mM ZnCl_2_ and our method. The results are shown on Figure 4. The HCCB has a viscosity close to water being a good starting point. The addition of 14% of PEG resulted in the highest viscosity increase, as expected. There is a considerable difference once the 2 mM of ZnCl_2_ is added to the mixture and the amount of PEG is decreased by half. As we can observe, there is half of the increase in the viscosity. Indicating that the PEG is the major contributor to the increase in the viscosity. In our method, since there is no PEG, the increase in the viscosity is negligible. As we stated earlier, this represents a great advantage when working with filtration units allowing the use of higher flow rates without substantially increasing the pressure drop and more importantly, minimizing the membrane fouling [24, 25].

### 3.4 Continuous capture

**FIGURE 5:**
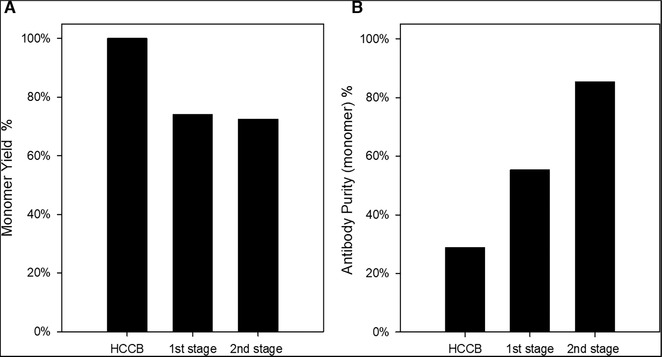
Continuous precipitation integrated with a tangential flow filtration (TFF). (A) Process yield and (B) purity of the antibody monomer: starting material before precipitation, host cell culture broth (HCCB); after antibody precipitation and concentration (first stage); and after the precipitate wash with continuous diafiltration (second stage)

We used tangential flow filtration (TFF) for the continuous separation and washing of the precipitate. Using Burgstaller et al. and Li et al. work as a starting point, we performed continuous precipitation integrated with a continuous separation using a two‐step TFF. The setup was tested using 1.3 L of HCCB containing Trastuzumab (2 g/L). The optimal precipitation conditions determined previously were used in this continuous setup without further modification. The HCCB feed stream was combined with the precipitant stream (ZnCl_2_) and a NaOH stream for pH adjustment in a specific ratio to achieve a final precipitation condition of 12 mM ZnCl_2_ pH 7. This was then pushed into a tubular reactor with static mixers that prevented the settling of the precipitate. The diameter and the length of the tubular reactor were taken into account and then combined with the flow rate to generate a residence time of approximately three minutes. This time was defined taking into account the experiments with the FBRM where one could observe that after the addition of the precipitant and the pH correction, it takes 2–3 min of mixing to achieve a solution with stable clusters. In the first stage, the precipitate was continuously concentrated with TFF (pore size 0.2 µm; membrane area 50 cm²). The precipitate retentate velocity (stream over the membrane) was 1440 L m^−2^ h^−1^ (LMH) as a starting point and was taken from Burgstaller [27] who used fluxes ranging from 571 LMH to 2182 LMH. The permeate flux was constant over time, 92.4 LMH, and the precipitate was concentrated to a factor of 10 and continuously withdrawn as a bleed from the reservoir of the TFF. The collected bleed was pooled and had a final volume of approximately 130 mL. In a second stage, the precipitates were washed with 50 mM Tris‐HCl pH 7 by diluting the precipitate harvest from the first step and using the same membrane and fluxes as before to achieve again a concentration factor of 10, resulting again in a final volume after the wash of 130 mL. The integrated set up ran for about 200 min to process the 1.3 L starting material, both feed pressure and transmembrane pressure (TMP) were constant during the whole run, 16 and 10 kPa, respectively. The overall process yield and purity are presented in Figure 5.

As can be seen, the overall process has a yield of approximately 73%. The major product loss was in the first stage, where incomplete precipitation was observed. Around 26% of Trastuzumab on the initial sample did not precipitate and was found in the permeate tank of stage 1, which is not in line with the batch experiments for Trastuzumab. A further investigation will be needed to understand what the cause of this loss is if it is related to the precipitation mechanism, residence time, shear forces, mixing behavior, or operating fluxes. There was no evidence pointing to membrane fouling, as the TMPs in the dewatering and wash remained stable during the process as was expected due to the low viscosity. Nonetheless, the TMP and feed pressures were higher than the ones reported by Li et al. as result from a higher feed and permeate flux but we do not think that this would lead to lower precipitation yields.

Regardless of the loss, once the precipitate was formed, it appeared to be stable on the running conditions also during the wash since no further loss was observed. During the first stage, the antibody relative purity increased from 28 to 55%. Further removal of impurities was achieved with the washing step. In addition, since no ZnCl_2_ was added to the washing buffer, besides washing out the impurities (high molecular weight impurities, host cell proteins, and DNA), in this section we could also remove the excess of ZnCl_2_ possibly facilitating the resolubilization process. The concentrated precipitate was washed with approximately three times its volume (around 500 mL).

**FIGURE 6:**
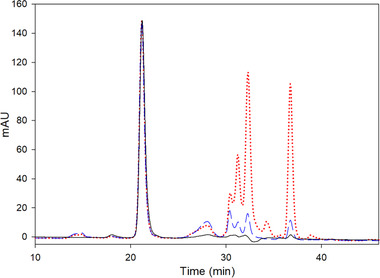
Typical size exclusion chromatogram: Red dotted line starting material before precipitation, host cell culture broth (HCCB), blue dashed line after antibody precipitation and concentration (first stage) and black solid line after the precipitate wash with continuous diafiltration (second stage)

At the end of the second stage, the relative monomer purity was around 85%, which is very comparable to the 89% or the 91% reported by Li et al. and Burgstaller et al., respectively, but with significantly lower dilution factors [4, 7]. Figure 6 shows the size exclusion chromatograms of samples at the end of each stage. Additionally, we evaluated even further the DNA and HCP clearance of the method. Our starting product had 1246 ± 174 µg/mL of HCP content and 5579 ± 223 ng/mL of DNA. In the end, we finished with a 1.3 L of purified Trastuzumab with a concentration of 1.48 g/L containing less than 2% of HMWI, 160 ± 11 µg/mL of HCP and 30 ± 3.6 ng/mL of DNA.

In the future, the optimization potential for this method is in the elucidation for the lower yield during continuous operation and the amount of washing buffer used in the second stage. Nevertheless, we were able to show the use of ZnCl_2_ as effective precipitating for antibody capture on its own without the addition of PEG, resulting in a low viscosity precipitate and low dilution factors in continuous operation.

## 4 CONCLUDING REMARKS

In this study, we showed that the precipitation of recombinant antibodies using ZnCl_2_ without other additives is possible and feasible. We studied three different antibodies trying to get a better understanding of the mechanism, requirements, limitations, and benefits of using ZnCl_2_ in the precipitation. The results point out that this cross‐linking agent has a higher affinity for proteins with higher number of histidines at its surface in agreement with previous publications. Even more, different IgG subclasses show different precipitation yields. We could establish that the pH was one of the major factors without being related to the pI, but even so, the differences in the precipitation yields between different antibodies were considerable. We speculated that the particular features such as the position of histidine amino acids within the antibody structure and the flexibility of the different IgGs may also play a role, but further investigations are needed to ensure this statement. Besides the yield of precipitation, the pH also shows to modulate the size distribution of the aggregates. The continuous precipitation studies showed results comparable to other recombinant antibodies precipitation studies, with the advantage of having a process that has the final product in a low dilution of a mildly low pH and a low conductivity that could possibly be fed into a continuous viral inactivation step. The resolubilization by pH shift to low pH neatly fits into the common scheme of antibody purification used in combination with protein A chromatography, which means that using this precipitation, the chromatography step can be exchanged without changing the overall downstream scheme.

## CONFLICT OF INTEREST

The authors have declared no conflict of interest.
